# Melatonin as a promising therapeutic intervention for restoring ovarian function in letrozole-induced polycystic ovary syndrome rats

**DOI:** 10.1016/j.heliyon.2023.e21237

**Published:** 2023-10-27

**Authors:** Muddasir Basheer, Aashaq Hussain Bhat, Younis Ahmad Hajam, Gaber El-Saber Batiha, Farid S. Ataya, Dalia Fouad, Seema Rai

**Affiliations:** a Department of Zoology, Guru Ghasidas Central University, Bilaspur, Chhattisgarh, 495009, India; b Department of Biosciences, University Centre for Development and Research, Chandigarh University, Gharuan, Mohali, Punjab, 140413, India; c Experimental Biology Research Group, Faculty of Science, University of Neuchatel, Neuchatel, Rue Emile Argland, 2000, Switzerland; d Department of Pharmacology and Therapeutics, Faculty of Veterinary Medicine, Damanhour University, Damanhour, 22511, AlBeheira, Egypt; e Department of Biochemistry, College of Science, King Saud University, PO Box 2455, Riyadh, 11451, Saudi Arabia; f Department of Zoology, College of Science, King Saud University, PO Box.22452, Riyadh, 11495, Saudi Arabia; g Department of Zoology, Government Degree College for Women, Anantnag, Jammu and Kashmir, 192101, India; h Department of Life Sciences and Allied Health Sciences, Sant Baba Bhag Singh University, Jalandhar, Punjab, 144030 India

**Keywords:** Melatonin, Steroidogenic enzymes, Follicle, Letrozole, PCOS

## Abstract

Polycystic ovary syndrome (PCOS) is a common hormonal disorder that affects women of reproductive age and is characterized by multiple ovarian cysts, irregular menstrual cycles, and excessive androgen hormone production. The present study aimed to investigate the therapeutic efficacy of melatonin in alleviating PCOS-induced alterations in female Wistar rats. PCOS was induced in female albino rats by administering letrozole at a dose of 1 mg/kg for 21 days. A total of 24 rats were randomly selected and divided into four groups: group I (normal control), group II (melatonin treatment), group III (letrozole treatment), and group IV (melatonin therapy for PCOS rats). Physical parameters (body and uterus weight), hormone profile (LH and FSH), and steroidogenic enzyme activities and an oral glucose test were assessed using standard methods. Histological analysis was performed using hematoxylin and eosin staining. The results demonstrated that exogenous melatonin administration significantly improved PCOS symptoms in rats, including reduced body weight gain, changes in organ weight/body weight index, blood glucose level, percentage diestrus phase, testosterone, estradiol, progesterone, and LH/FSH ratio, as well as 3β-HSD and 17β-HSD enzyme activity. Histopathological findings revealed well-developed follicles, decreased cystic follicles, and increased antral follicles, Graafian follicles, and corpus luteum in PCOS rats treated with melatonin. These positive outcomes suggest that exogenous melatonin may hold promise as a valuable remedy for PCOS conditions in female rats. Further research is warranted to fully elucidate the underlying mechanisms and potential clinical applications of melatonin in the context of PCOS.

## 1 Introduction

Polycystic ovary syndrome (PCOS) is a condition that affects the reproductive and endocrine systems of women during their reproductive years. It is caused by an imbalance of steroidal hormones, leading to problems with ovarian follicular development and ovulation. This disorder is widespread and affects around 8–13 % of women in their reproductive age [1]. PCOS not only impacts reproductive health, but also effects on the endocrine and metabolic systems [2]. It increases the risk of developing health issues such as high blood pressure, diabetes mellitus, cardiovascular disease, and endometrial cancer. PCOS manifests physically with hirsutism (excess facial and body hair), severe acne, and obesity [3]. The primary characteristic of this syndrome is elevated levels of androgens, which are further exacerbated by the circulation of excess ovarian androgen production from multiple small follicles, anovulation, and insulin resistance in women's reproductive life [[4], [5]]. Therefore, elevated androgen levels are the main marker of PCOS [5].

PCOS was first described by Stein and Leventhal in the mid-1930s, but the exact cause of this disorder remains unclear [6]. Although several factors are involved, most treatments for PCOS aim to alleviate its symptoms and cannot completely cure it [7]. Currently available treatments for PCOS are still a topic of investigation and debate among researchers, as many proposed drugs are reported to have various side effects with prolonged usage [8,9]. These medications aim to regulate the reproductive cycle and stimulate ovulation. Managing PCOS effectively remains a challenge because different drugs are used to address different symptoms of the disorder.

Melatonin, also known as *N*-acetyl-5-methoxytryptamine, is a neurohormone primarily synthesized and released by pinealocytes in mammals rhythmically [10]. Its production and release increase during darkness and decrease during light. While the pineal gland is the primary source of melatonin, approximately 25 % of its secretion comes from sources outside the gland [11]. Melatonin is involved in several physiological processes, including the regulation of reproduction and circadian rhythms [[12], [13], [14]]. Recent studies have revealed that melatonin is a powerful scavenger of various reactive oxygen species (ROS), such as hydroxyl and peroxyl radicals, and is known for its immune-modulatory effects and anti-aging properties [14,15]. This neurohormone with a hydrophilic and lipophilic structure can easily cross all biological barriers and reach a high concentration in intracellular components, where it directly neutralizes free radicals, such as reactive oxygen species (ROS), and prevents cellular damage caused by oxidative stress [16,17]. It enhances the activity of antioxidant enzymes, further bolstering the body's defense against oxidative damage. As a powerful antioxidant and free radical scavenger, melatonin has multiple benefits for reproduction, such as antioxidative stress in germ cells, delay of ovarian aging, promotion of oocyte maturation, and augmentation of embryo development. In the ovaries, it scavenges free radicals and protects ovarian cells from oxidative damage. By neutralizing harmful reactive species, melatonin helps maintain the health and proper functioning of ovarian tissues, supporting female reproductive processes and potential fertility [[18], [19], [20]].

Due to ethical considerations, studying PCOS in human subjects is limited. Animal models of PCOS provide a valuable tool for investigating the underlying pathogenesis and metabolic and endocrinological influences, and for developing potential therapies. Various methods have been employed to induce PCOS-like conditions in rat models, including exposure to androgens, estrogens, constant light, or genetic modifications [21]. These models have demonstrated some features of PCOS. Letrozole, a non-steroidal aromatase inhibitor that blocks the conversion of androgens to estrogens, can induce several reproductive, endocrine, and metabolic abnormalities, including anovulation, ovarian morphology and histoarchitecture changes, insulin resistance, and abnormal hormone levels in female rats, making it a suitable model for studying PCOS pathology and treatment options [22,23]. In human females, insulin resistance is prevalent in 50–70 % of cases and is associated with PCOS. Insulin resistance and high insulin levels are important factors in the development of PCOS, leading to metabolic complications and cardiovascular disorders [[24], [25], [26], [27]]. PCOS also affects blood vessel function and other blood properties, further worsening the condition. Patients with PCOS often experience oxidative stress and inflammation, which can affect blood cells [28,29]. The viscosity of whole blood increases during PCOS due to elevated blood plasma viscosity [30]. Therefore, this study was planned to investigate the effects of external melatonin supplementation on letrozole-induced PCOS in Wistar rats.

Given the promising antioxidant and regulatory effects of melatonin on hormonal balance, we aimed to investigate the potential effects of external melatonin supplementation in letrozole-induced PCOS in female Wistar rats. We explored the impact of melatonin treatment on blood glucose levels, body weight, estrous cycle, hormone profiles, ovarian and uterine weights, and steroidogenic enzyme activities. Additionally, we assessed its effect on ovarian histopathology and folliculogenesis to gain insights into its impact on reproductive processes and potential fertility. The findings of this study may provide valuable insights into the role of melatonin as a therapeutic agent for managing PCOS-related complications and hormonal imbalances. Understanding the potential benefits of melatonin in improving reproductive health in PCOS could pave the way for developing novel treatment strategies for this prevalent and complex hormonal disorder.

## 2 Materials and methods

### 2.1 Chemicals and reagents

The chemicals and reagents utilized in the study were of analytical grade and were procured from HiMedia Laboratories Pvt. Ltd. Mumbai, India and Sisco Research Ltd. (SRL). Letrozole and melatonin were purchased from Novartis India Ltd. and Sigma-Aldrich, USA, respectively. ELISA kits were employed for the analysis of serum hormones, and the manufacturer's instructions were strictly followed during the analysis.

### 2.2 Animal maintenance

The experiments were conducted following the guidelines of the revised Animals (Specific Procedure) Act of 2002 of the Government of India for animal welfare and were approved by the Institutional Animal Ethics Committee (IAEC) of Guru Ghasidas Vishwavidyalaya (Registration Number: 994/Go/ERe/S/06/CPCSEA) Bilaspur, Chhattisgarh, India. Adult female Wistar albino rats of the same age and weighing approximately 190 ± 10 g were procured from Defence Research and Development Establishment (DRDE) Gwalior, M.P. India and acclimatized under standard husbandry conditions (25 ± 2 °C, 60–70 % relative humidity, and 12 h photoperiod) with access to standard rat feed and drinking water *ad libitum*. The experimental procedures were approved by the Committee for the Purpose of Control and Supervision of Experiments on Animals (CPCSEA) under the Institutional Animal Ethics Committee at SLT Institute of Pharmaceutical Sciences, Guru Ghasidas Vishwavidyalaya, Bilaspur, Chhattisgarh, India. The experimental groups were randomly divided into four groups, each containing six rats, and named and numbered as per the experimental design.

### 2.3 PCOS induction

To induce PCOS, a dosage of 1 mg/kg of letrozole dissolved in 1 % Carboxy Methyl Cellulose (CMC) was administered daily for 21 days. The control group was given only 1 % CMC. The induction was confirmed by collecting vaginal smears daily and evaluating them microscopically [13].

### 2.4 Experimental design

The study involved 24 female rats, which were divided into four groups: Control (CON), Melatonin (MEL), Letrozole (PCOS), and PCOS + Melatonin. Group I (CON) received 1 % Carboxy Methyl Cellulose (CMC) solution, Group II (MEL) received 1 mg/kg body weight of melatonin orally for three weeks, Group III (PCOS) received 1 mg/kg body weight of letrozole orally for three weeks, and Group IV (PCOS + MEL) received 1 mg/kg body weight of letrozole and 1 mg/kg body weight of melatonin orally for three weeks. The oral administration of melatonin was done during the evening time. The rats were monitored for any adverse effects during the study period. At the end of the treatment period, their ovaries were collected for analysis to determine the effect of melatonin supplementation on ovarian function in rats with letrozole-induced polycystic ovary syndrome.

### 2.5 Oral glucose tolerance test

For the glucose tolerance test, rats were fasted for 12 h before being administered glucose at a rate of 300 mg/kg of body weight. Blood samples were taken from the rats' tail veins at 30 min intervals for 2 h following the administration of glucose. The blood samples were then tested for glucose levels using a glucometer (Accu-check, Roche Diabetes Care, India), as described by Goto et al. [31].

### 2.6 Animal sacrifice & sample collection

Animals were weighed at the beginning and end of the experiment. After the experimental period ended, all the rats were sacrificed using cervical dislocation while under diethyl ether anesthesia. Blood samples were taken from the heart through a cardiac puncture and stored in vials coated with EDTA. The blood was then centrifuged at 3000×*g* to separate the serum, which was stored at −20 °C for later enzyme-linked immunosorbent assays (ELISA). The ovaries and uteruses were taken out, washed in normal saline, and weighed.

### 2.7 Steroidogenic enzymes assay

#### 2.7.1 Preparation of ovarian homogenate

At the end of the experiment, the animals were euthanized, and their ovaries were extracted. The ovaries were weighed, and a 10 % ovarian tissue homogenate was prepared using 0.1 M Tris HCl buffer (with a pH of 7.8). The homogenate was then centrifuged at 80,000×*g* for 10 min at a temperature of 40 °C. The resulting supernatant was collected in a tube for use in a steroidogenic enzyme assay. The amount of protein in the supernatant was determined using the method described by Lowry et al. [32].

#### 2.7.2 3β-hydroxysteroid dehydrogenase activity assay

The activity of 3β-hydroxysteroid dehydrogenase (3β-HSD) in the ovarian homogenate was measured using the method of Shivanandappa and Venkatesh [33]. The assay was conducted in a 0.1 M Tris HCl buffer (pH 7.8) containing 500 μM nicotinamide adenine dinucleotide (NAD^+^) and 100 μM dehydroepiandrosterone (DHEA) substrate for 3β-HSD in a total volume of 3 mL. Enzyme reaction was initiated by adding 100 μL ovarian homogenate and the color reagent iodonitrotetrazolium (INT). The reaction mixture was incubated at 37 °C for 1 h and terminated by adding 2.0 mL phthalate buffer (pH 3.0). The absorbance was then measured at 490 nm. The enzyme activity was determined using the standard curve for NADH and expressed as nmol of NADH formed/min/mg of protein.

#### 2.7.3 17b- HSD enzyme activity assay

The enzymatic activity of 17β-HSD was evaluated using the Bergmeyer method [34], which involved measuring the rate of conversion of NADPH to NADP by optical means. To carry out this assay, a reaction mixture was prepared consisting of 100 μL of supernatant from ovarian homogenate, 200 μL of 0.5 μM NADPH, 100 μL of 0.8 μM androstenedione, and 3 mL of 100 μM phosphate buffer solution (pH 7.4). The reaction was initiated by adding the substrate and the absorbance of NADPH was recorded at 340 nm for 5 min, at 20 s intervals. The enzyme activity was expressed as nmol of NADPH oxidized/minute/milligram of protein.

#### 2.7.4 Hormone assays

The serum samples collected from each group of female rats were stored for future hormonal ELISA analysis of luteinizing hormone (LH) and follicle-stimulating hormone (FSH) using kits (Monobind Inc., Costa Mesa, USA). Commercial kits (ELISA kit, Amegnix, Inc., Burlingame, CA, USA) were used to measure testosterone, estradiol, and progesterone, following the instructions provided in the catalog. The UV/Visible spectrophotometer (PerkinElmer, USA) was used for spectrophotometric analysis. The intra- and inter-assay coefficient of variance (CV) and recovery values were 5.5 %, 8.5 %, and 92 %, respectively.

#### 2.7.5 Histopathological analyses

The ovaries were washed with normal saline and then fixed with Bouin's fixative. After fixation, the ovaries from each experimental group were dehydrated using graded ethanol series. The ovarian tissue was then cleared with xylene and embedded in paraffin wax to prepare blocks. The ovary embedded within the blocks was sliced into ribbons/sections, which were 4–5 μm thick, using a rotary microtome (Leica RM 2125-RT 5). The sections were processed for staining using hematoxylin and eosin, mounted with DPX, and viewed under a light microscope (Magnus, India). Follicles at different stages, including primary, preantral, antral, Graafian, cystic and corpus luteum, were identified and counted. Morphometric measurements were taken using the Nikon DS-L1 image acquisition software mounted on a phase-contrast microscope (Nikon Eclipse 50i) in μm.

### 2.8 Statistical analysis

The statistical data analysis was conducted using SPSS 16.0 software (SPSS, Chicago, IL, USA). Multiple comparisons were assessed using Student's t-tests, with a significance level set at p ≤ 0.05 and p ≤ 0.01. The results are presented as the Mean ± Standard Error of the Mean (SEM) [35].

## 3 Results

### 3.1 Effect of melatonin on blood glucose level in PCOS rats

There was no statistically significant difference observed in the blood glucose levels between the control group and the rats treated with melatonin. However, the rats with PCOS exhibited elevated glucose levels, which were significantly reduced after treatment with melatonin (Table 1). The observed reduction in blood glucose levels after melatonin treatment indicates its potential as a therapeutic agent to counteract hyperglycemia in PCOS and highlights the importance of managing glucose homeostasis in individuals with this condition. Melatonin administration had a beneficial impact on glucose regulation, potentially enhancing insulin sensitivity and/or improving glucose uptake by cells. By lowering blood glucose levels, melatonin helps mitigate the insulin resistance associated with PCOS, reducing the risk of long-term complications related to hyperglycemia.

**Table 1:** Effect of exogenous melatonin in PCOS rat model with respect to glucose and hormone parameters in groups.

Groups	Glucose (mg/dl)	Testosterone (ng/mL)	Progesterone (ng/mL)	Estradiol (pg/mL)	LH (mIU/ml)	FSH (mIU/ml)	LH/FSH
Control (CON)	200 ± 8	3.87 ± 0.53	29.54 ± 1.56	28.7 ± 1.42	31.62 ± 2.14	32.31 ± 2.32	**0.9** ± 0.23
PCOS	300 ± 11**	10.23 ± 0.95**	12.57 ± 2.32**	14.63 ± 1.25**	16.43 ± 1.69**	17.47 ± 1.58**	1.7 ± .42 **
PCOS + MEL	222 ± 6**	4.61 ± 0.63**	16.59 ± 2.14*	30.20 ± 0.96**	29.88 ± 2.45**	28.25 ± 3.21**	1.1 ± 0.33**
MEL	220 ± 10**	4.15 ± 0.45**	20.25 ± 1.96**	31.52 ± 1.21**	32.96 ± 2.54**	33.12 ± 52**	1.00 ± 0.15**

Data are Mean ± SEM; N = 6. PCOS vs CON, PCOS vs PCOS + MEL, PCOS vs MEL; Superscripts denotes; *p < 0.05; **p < 0.01; ***p < 0.001.

### 3.2 Effect of melatonin on body weight in PCOS rats

**Fig. 1:**
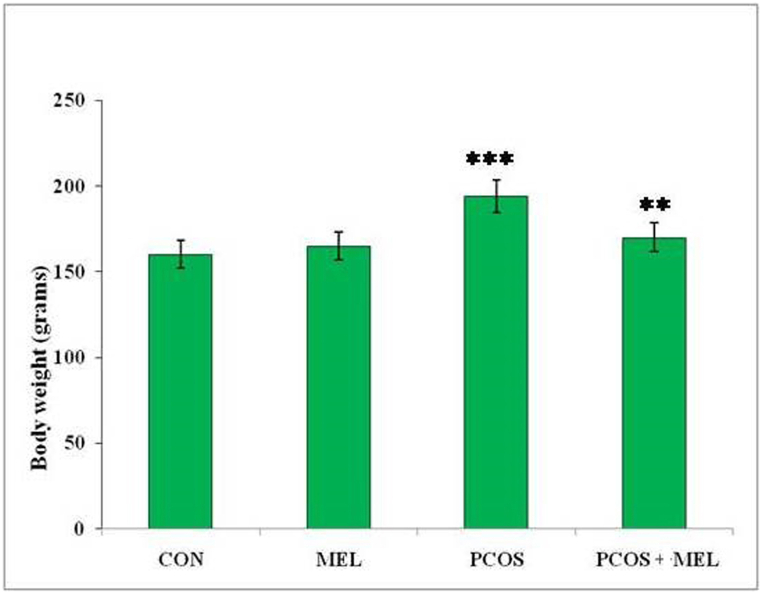
Effect of exogenous melatonin on the body weight of different experimental groups of Letrozole-induced female PCOS rats. Histogram represents Mean ± SE; N = 6. CON = Control, PCOS = Polycystic ovary syndrome, MEL = Melatonin. PCOS vs CONT. PCOS vs PCOS + MEL, ***P ≤ 0.001, **P ≤ 0.01.

The administration of letrozole resulted in a significant increase in the body weight of rats compared to the control group. The significant increase in body weight observed in the rats treated with letrozole indicates that the induction of PCOS is associated with weight gain. Letrozole is known to interfere with estrogen synthesis, leading to alterations in hormonal balance and metabolic changes, which may contribute to weight gain in this model. Conversely, treating PCOS rats with melatonin lead to a decrease in their body weight, which was found to be significantly similar to the control group (Fig. 1). However, it is important to note that despite the reduction in body weight, the values were still significantly similar to the PCOS group, implying that melatonin did not completely reverse the weight gain associated with PCOS.

### 3.3 Effect of melatonin on estrous cycle in PCOS rats

**Fig. 2:**
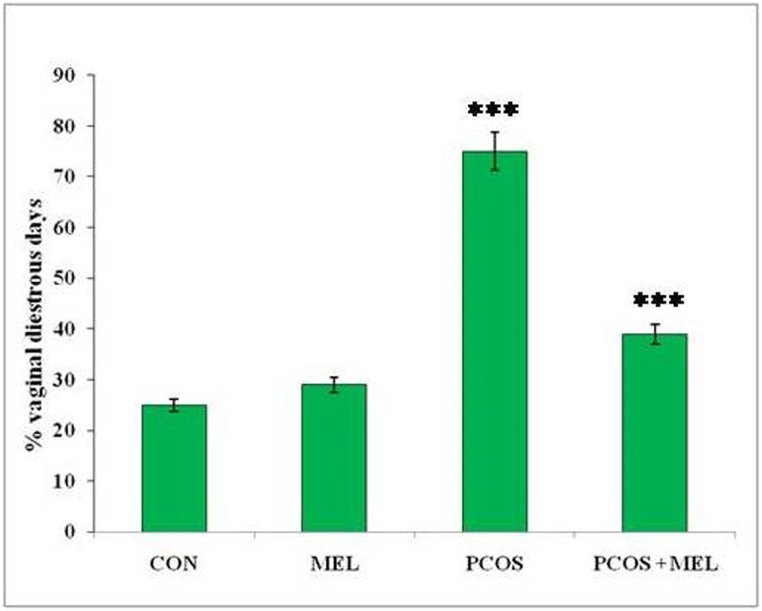
Effect of exogenous melatonin on the percent diestrus days in Letrozole-induced female PCOS rats. Histogram represents Mean ± SE; N = 6. CON = Control, PCOS = Polycystic ovary syndrome, MEL = Melatonin. PCOS vs CONT. PCOS vs PCOS + MEL, * **P ≤ 0.001.

Female rats undergo a reproductive cycle of 4–5 days, which includes four phases: proestrus, estrous, metestrus, and diestrus phases. Measuring the reproductive cycle in rats is crucial for understanding their hormonal and reproductive health. In this study, PCOS rats exhibited a prolonged diestrus phase leading to a significantly higher percentage of days spent in diestrus compared to the control group. This prolonged diestrus phase indicates a disruption in their reproductive cycle, potentially linked to hormonal imbalances and ovarian dysfunction associated with PCOS. Such an extended diestrus phase can affect the normal hormonal fluctuations required for successful ovulation and other reproductive processes. However, the administration of melatonin to PCOS rats resulted in a significant reduction in the percentage of diestrus days, similar to the control group (Fig. 2). This finding suggests that melatonin treatment helps restore the normal duration and distribution of the reproductive cycle phases in PCOS rats. Melatonin's regulation of hormonal imbalances and improvement of ovarian function may contribute to a more normalized reproductive cycle.

### 3.4 Effect of melatonin on serum hormone profile in PCOS rats

Measuring the testosterone, progesterone, and estradiol levels in rats is crucial for understanding their hormonal status and reproductive health, as these hormones play essential roles in regulating the reproductive system and other physiological processes. In the current study, we observed significant changes in these hormone levels in the PCOS-induced group compared to the control group. Specifically, the testosterone level was significantly increased in the PCOS-induced group (P < 0.001) indicating the presence of hyperandrogenism, a common feature of PCOS. Conversely, progesterone and estradiol were significantly decreased in the PCOS group, suggesting a disruption in the normal hormonal balance. Melatonin treatment was able to restore the hormonal values near the control, suggesting that it had a beneficial effect on hormonal regulation in the context of PCOS. These alterations in hormone levels are characteristic of PCOS and may contribute to the reproductive and metabolic issues observed in affected individuals.

Melatonin treatment was found to have a beneficial effect on hormonal regulation in the context of PCOS. It was able to restore the testosterone, progesterone, and estradiol levels near the control group values, suggesting its potential as a therapeutic agent for managing hormonal imbalances associated with PCOS. By restoring these hormone levels, melatonin may help alleviate some of the symptoms and complications of PCOS, potentially improving reproductive health and overall well-being in affected rats. In addition to hormonal imbalances, PCOS can also disrupt the normal pattern of follicle-stimulating hormone (FSH) and luteinizing hormone (LH) secretion, affecting ovulation and ovarian function. In the current study, rats treated with letrozole exhibited a marked reduction in FSH levels compared to control group. However, with melatonin treatment, there was a significant recovery in serum FSH levels, indicating its potential to regulate FSH secretion and restore ovarian function in the context of PCOS. The LH level was significantly elevated in the PCOS group, and we calculated the LH/FSH ratio based on the value of LH and FSH. The LH/FSH ratio was significantly elevated in the PCOS group, indicating an imbalance between LH and FSH secretion, which can disrupt ovulation and contribute to the development of ovarian cysts. Importantly, melatonin treatment effectively regulated and restored the LH/FSH ratio (Table 1), further emphasizing its potential in improving hormonal balance and managing PCOS-related reproductive issues.

### 3.5 Effect of melatonin on ovarian, uterine & organ weight/body weight index in PCOS rats

**Fig. 3:**
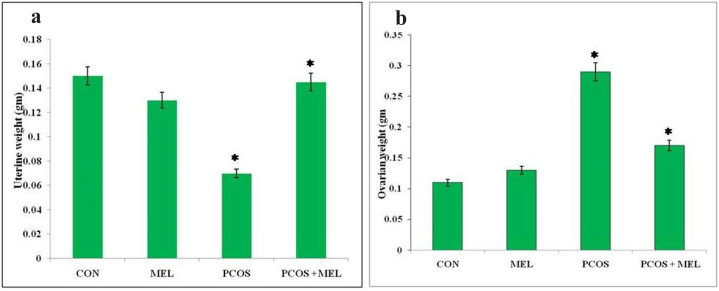
Effect of exogenous melatonin on the (a) uterine weight, and (b) ovarian weight of Letrozole-induced female PCOS rats. Histogram represents Mean ± SE; N = 6. CON = Control, PCOS = Polycystic ovary syndrome, MEL = Melatonin. PCOS vs CONT. PCOS vs PCOS + MEL, *P ≤ 0.05.

**Fig. 4:**
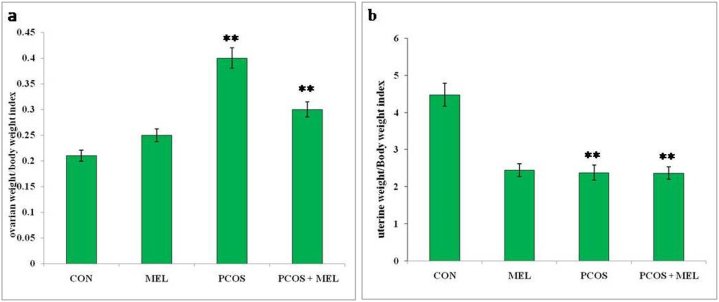
Effect of exogenous melatonin on the (a) ovarian weight/body weight index, and (b) uterine weight/body weight index of Letrozole-induced female PCOS rats. Histogram represents Mean ± SE; N = 6. CON = Control, PCOS = Polycystic ovary syndrome, MEL = Melatonin. PCOS vs CONT. PCOS vs PCOS + MEL, **P ≤ 0.01.

In rats with polycystic ovary syndrome (PCOS), there was a significant increase in the ratio of ovary weight to body weight compared to the control group, indicating an enlargement of the ovaries relative to body size. Conversely, the PCOS group of rats exhibited a decrease in the ratio of uterine weight to body weight compared to the control group, suggesting a reduction in uterine size relative to body weight. However, treatment with melatonin in PCOS rats led to a significant reduction in the ratio of ovary weight to body weight, indicating that melatonin intervention effectively counteracted the ovarian enlargement observed in PCOS. On the other hand, melatonin treatment did not significantly impact the ratio of uterine weight to body weight when compared to the PCOS group, suggesting that the uterine size remained relatively unchanged by melatonin administration (Fig. 3a and b and Fig. 4a and b). The measurement of ovary and uterus weight and their respective indexes provides valuable insights into the reproductive changes occurring in PCOS and offers a means to assess the efficacy of interventions, such as melatonin treatment, in modulating these reproductive parameters. This information is crucial for understanding the pathophysiology of PCOS and evaluating potential therapeutic strategies to manage this condition.

### 3.6 Effect of melatonin on steroidogenic enzymes (3β-HSD and 17β-HSD) in PCOS rats

**Fig. 5:**
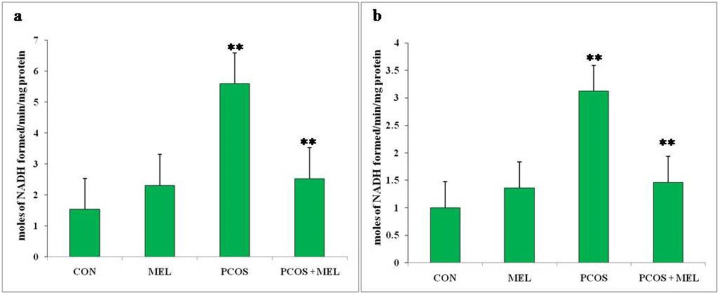
Effect of exogenous melatonin on a) *3β-HSD*, and (b) *17β-HSD* steroidogenic enzyme activity of Letrozole-induced female PCOS rats. Histogram represents Mean ± SE; N = 6. CON = Control, PCOS = Polycystic ovary syndrome, MEL = Melatonin. PCOS vs CONT. PCOS vs PCOS + MEL, **P ≤ 0.01.

Measuring the levels of 3β-hydroxysteroid dehydrogenase (3β-HSD) and 17β-hydroxysteroid dehydrogenase (17β-HSD) in rats is essential for understanding the enzymatic activities involved in steroid hormone synthesis and metabolism. These enzymes play crucial roles in the conversion of steroid hormones, including androgens and estrogens, which are involved in regulating various physiological processes, including reproductive function. in the current study, the levels of 3β-HSD and 17β-HSD were found significantly higher (p < 0.01) in PCOS rats compared to the control rats, which indicates an upregulation of these enzymatic activities. This upregulation is likely contributing to the higher production and accumulation of steroid hormones, such as androgens and estrogens, which are associated with PCOS. Elevated androgen levels are a hallmark of PCOS and can lead to symptoms like hirsutism, acne, and irregular menstrual cycles. Additionally, imbalances in estrogen levels can further disrupt the hormonal environment and contribute to fertility issues. Administering melatonin to the PCOS rats resulted in a considerable reduction in the levels of 3β-HSD and 17β-HSD compared to PCOS rats (Fig. 5a and b). This reduction in enzymatic activity indicates that melatonin treatment has a regulatory effect on steroid hormone synthesis and metabolism. By decreasing the activities of 3β-HSD and 17β-HSD, melatonin may help normalize the production and metabolism of androgens and estrogens, restoring the hormonal balance disrupted in PCOS. The decrease in 3β-HSD and 17β-HSD levels observed with melatonin treatment in this suggests that it may be a potential therapeutic agent for managing the hormonal imbalances associated with PCOS. By modulating these enzymatic activities, melatonin could help alleviate the symptoms and complications related to excess androgens and disrupted estrogen levels in PCOS.

### 3.7 Effect of melatonin on histopathology and folliculogenesis in PCOS rats

**Fig. 6:**
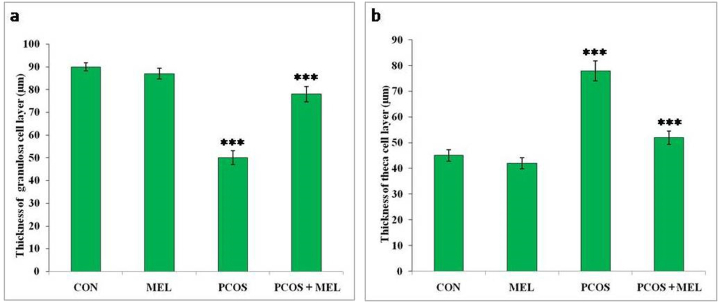
Effect of exogenous melatonin on the thickness of (a) granulose cell layers, and theca cell layers of Letrozole-induced female PCOS rats. Histogram represents Mean ± SE; N = 6. CON = Control, PCOS = Polycystic ovary syndrome, MEL = Melatonin. PCOS vs CONT. PCOS vs PCOS + MEL, ***P ≤ 0.001.

**Fig. 7:**
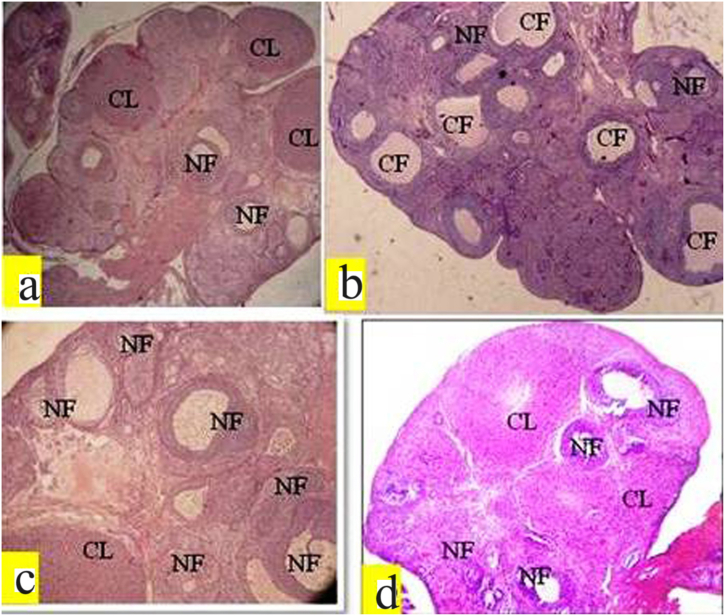
Histomicrograph of PCOS rat's ovary showing exogenous melatonin effect. (a) Control (CON) group exhibiting normal architecture. (b) Letrozole (LET) treatment caused the formation of cystic follicles, and decreased the number of different categories of follicles. Thickness and diameter of theca layer in the PCOS group increased significantly, while as diameter and thickness of granulose cells decreased significantly compared to the control group of rats. (c) Melatonin (MEL) administration maintained the normal ovarian structure. (d) Letrozole (LET) + Melatonin (MEL) administration restored the various parameters of folliculogenesis towards normal. CF: Cystic follicle, NF: normal follicle, CL: corpus luteum.

Measuring the follicles and corpus luteum in rats provides valuable insights into the ovarian folliculogenesis process and its alterations in the context of PCOS and melatonin treatment. In the current study, the PCOS group of rats showed a considerable increase in the cystic follicles compared to both the control and melatonin treatment groups (Table 2, Table 3, and Fig. 7a–d). This increase in cystic follicles indicates a disruption in the normal folliculogenesis process, which may contribute to the reproductive issues observed in PCOS. However, PCOS rats treated with melatonin after induction exhibited a significant increase in the number of preantral, antral, graafian follicles, and corpus luteum. Additionally, the thickness and diameter of the theca layer increased significantly in the PCOS group, while the diameter and thickness of granulose cells decreased significantly compared to the control group of rats. Treatment with exogenous melatonin effectively reversed these folliculogenesis parameters towards the control group (Fig. 6a and b). These findings suggest that melatonin may have a positive impact on ovarian function and hormonal regulation in PCOS rats. By restoring the balance in follicular development and ovulation, melatonin may help improve fertility outcomes in PCOS. The beneficial effects of melatonin observed in the present study indicate its potential as a therapeutic agent for managing ovarian dysfunction and hormonal imbalances associated with PCOS. These results contribute to our understanding of the mechanisms through which melatonin may influence reproductive health and offer promise for developing novel treatment approaches for PCOS-related fertility issues.

**Table 2:** Effect of exogenous melatonin in PCOS rat model with respect to mean follicle numbers in different groups.

Groups	Primary follicle	Preantral follicle	Antral follicle	Graafian follicle	Cystic follicle	Corpus luteum
Control (CON)	18.41 ± 0.32	50.24 ± 1.23	10.54 ± 0.90	1.52 ± 0.62	0	9.21 ± 0.19
PCOS	18 ± 0.65	37.25 ± 1.54***	3.21 ± 0.21***	0	5.86 ± 0.56***	3.12 ± 0.81***
PCOS + MEL	17.21 ± 0.60	47.59 ± 0.86**	6.54 ± 0.32**	1.88 ± 0.23	1.39 ± 0.47	7.21 ± 0.52
MEL	17.96 ± 0.82	52.21 ± 1.21	9.26 ± 0.41	1.43 ± 0.41*	1.01 ± 0.35**	8.25 ± 0.74**

Data are Mean ± SEM; N = 6. PCOS vs CON, PCOS vs PCOS + MEL, PCOS vs MEL; Superscripts denotes; *p < 0.05; **p < 0.01; ***p < 0.001.

**Table 3:** Effect of exogenous melatonin in PCOS rat model with respect to antral follicle morphometry in different groups.

Groups	Antral follicle diam. (μm)	Thickness of granulosa layer (μm)	Thickness of the theca layer (μm)
Control (CON)	362 ± 0.54	55.36 ± 0.26	19.32 ± 0.25
PCOS	570.21 ± 0.45***	30.21 ± 0.34**	46.21 ± 0.39**
PCOS + MEL	460.45 ± 0.63**	39.54 ± 0.47**	22.34 ± 0.93**
MEL	372.65 ± 1.12**	52.25 ± 0.78***	26.85 ± 0.77**

Data are Mean ± SEM; N = 6. PCOS vs CON, PCOS vs PCOS + MEL, PCOS vs MEL; Superscripts denotes; ***p < 0.001; **p < 0.01.

## 4 Discussion

Polycystic ovarian syndrome (PCOS) is a growing concern causing infertility in females, particularly during their reproductive age. In the present study on female rats, it was observed that melatonin treatment resulted in regularity in the reproductive cycle as opposed to the irregularity observed in the untreated rats. This regularity restoration is an indication of a return to normalcy. The changes in cyclicity observed in the melatonin-treated rats can be attributed to the alterations in sex hormones and gonadotropins, which significantly impact ovarian function. During PCOS conditions, the LH/FSH ratio is disturbed and can become two or three times more than the normal ratio of 1:1 [36]. Our studies also found that the LH/FSH ratio of gonadotropins was altered upon induction of the PCOS condition, which was restored towards the normal value with melatonin treatment. Our results were consistent with prior studies [37,38]. In PCOS rats, the estrogen levels were significantly elevated, which is the leading cause of anovulatory conditions. This leads to increased LH levels and a progesterone deficiency, forming cystic follicles and infertility. However, melatonin therapy caused a significant increase in FSH and a decrease in estrogen compared to the PCOS group, restoring the LH/FSH ratio to normal. Consequently, there was a considerable increase in the number of different follicles and corpora lutea. Our findings are consistent with other studies [37,39].

PCOS is not only a reproductive disorder but also a metabolic disorder that is linked to diabetes mellitus [40]. It can lead to hyperglycemia in the early stages, gradually progressing to insulin resistance or impaired glucose tolerance, resulting in elevated circulating glucose levels. If left uncontrolled, these elevated blood glucose levels, can have detrimental effects on various organs and tissues. In this study, rats with induced PCOS exhibited a significant increase in blood glucose levels, consistent with previous studies reporting the induction of hyperglycemia in rats with letrozole-induced PCOS [40,41]. However, after melatonin treatment, a significant reduction in blood glucose levels was observed after melatonin treatment in PCOS rats. Melatonin administration appeared to regulate glucose homeostasis, potentially enhancing insulin sensitivity and/or improving glucose uptake by cells. As a result, melatonin effectively lowered blood glucose levels, and helped mitigate the insulin resistance associated with PCOS, thereby reducing the risk of long-term complications related to hyperglycemia [40,41]. Inducing PCOS in rats with letrozole resulted in a significant increase in the activity of steroidogenic enzymes. However, the administration of melatonin therapy helped restore these enzyme levels to normal, which is consistent with findings from other studies [42,43]. Additionally, the PCOS group of rats in this study showed a significant increase in serum testosterone levels compared to the control group. This increase in testosterone levels is likely due to the inhibition of aromatase by letrozole, which prevents the conversion of androgens into estrogens and accumulates of androgens in the blood. The elevated testosterone levels in PCOS rats may contribute to the prolonged diestrus phase and weight gain. These findings are in agreement with previous studies [44,45].

Measuring the ovary and uterus weight in rats with polycystic ovary syndrome (PCOS) provides valuable information about the reproductive health of the animals and the impact of treatments. The index of ovary weight to body weight indicates the relative size and weight of the ovaries compared to the overall body weight. A notable rise in this index in PCOS rats suggests an enlargement or hypertrophy of the ovaries, which is a characteristic feature of PCOS and can indicate disrupted ovarian function. On the other hand, the index of uterine weight to body weight reflects the relative size and weight of the uterus compared to the overall body weight. The decrease in the uterine index in the PCOS group of rats suggests a reduction in uterine size, which could be due to hormonal imbalances and alterations in the reproductive system associated with PCOS. The study observed significant variations in the ovarian and uterine weights across various experimental groups. Specifically, rats with PCOS demonstrated a significant increase in ovarian weight and a substantial decrease in uterine weight. When melatonin was administered as a treatment to PCOS rats, it resulted in a significant reduction in the index of ovary weight to body weight. This decrease indicates that melatonin may have a positive effect in reducing the enlargement or hypertrophy of the ovaries associated with PCOS, potentially restoring more normal ovarian function. However, melatonin did not produce a noticeable effect on the index of uterine weight to body weight, suggesting that its administration may not have a significant impact on uterine size in the context of PCOS. These outcomes are consistent with similar previous research studies [[46], [47], [48]].

The study observed histological changes in the ovaries of rats with PCOS, including the formation of cysts, hyperplasia of internal theca cells, and thickening of the ovarian capsule. These changes were attributed to biologically active levels of FSH, increased LH, and lack of interplay between granulosa and theca cells [46,47]. Treatment with melatonin reduced ovary weight in rats treated with letrozole, potentially due to its ability to decrease testosterone levels. The melatonin treatment also showed protective effects in the ovaries, with well-developed antral follicles, a normal granulosa cell layer, and a defined theca layer. The number of atretic and cystic follicles in PCOS rats decreased significantly with melatonin treatment. Additionally, exogenous melatonin showed a significant reversal and restoration of histological changes in ovaries, with a reduction in cystic follicles and an increase in preantral and Graafian follicles and corpus luteum, likely due to its antioxidant effects [46,48]. Overall, the study's histological analysis indicates that melatonin may protect follicular development in rats with PCOS.

## 5 Conclusion

The current study highlights the therapeutic potential of melatonin in managing various aspects of polycystic ovary syndrome (PCOS) in rats. Melatonin administration effectively reduced elevated blood glucose levels, addressing hyperglycemia and emphasizing the importance of glucose homeostasis management in PCOS. Additionally, melatonin mitigated weight gain associated with PCOS induction, indicating its role in metabolic regulation. The restoration of the disrupted estrous cycle in PCOS rats further supports melatonin's impact on hormonal balance and ovarian function. Melatonin treatment also showed promising results in restoring serum hormone levels, particularly testosterone, progesterone, and estradiol, contributing to the alleviation of PCOS symptoms. Moreover, melatonin's regulation of steroidogenic enzyme activity suggests its potential in restoring hormonal balance and managing androgen and estrogen imbalances characteristic of PCOS. Notably, melatonin exhibited positive effects on ovarian folliculogenesis, enhancing the development of preantral, antral, graafian follicles, and corpus luteum, thereby improving fertility outcomes. These findings provide valuable insights into the mechanisms through which melatonin may ameliorate PCOS-related reproductive and metabolic issues and support its consideration as a promising therapeutic agent for PCOS management. Future studies are warranted to explore melatonin's long-term effects and its potential clinical application in human PCOS patients.

## Ethics statement

This study was approved by the Ethics Committee of the Guru Ghasidas Vishwavidyalaya (Registration Number: 994/Go/ERe/S/06/CPCSEA) (Reference No. 155/IAEC/Pharmacy/2016).

## Funding

The authors extend their appreciation to Researchers Supporting Project number (RSPD2023R-965), King Saud University, Riyadh, Saudi Arabia. The financial support of DBT BUILDER, Department of Biotechnology, Ministry of Science and Technology [grant number-BT/PR-7020/INF22/172–2012] is highly acknowledged.

## Author contribution statement

All authors listed have significantly contributed to the development and the writing of this article.

## Data availability

The datasets generated during and/or analyzed during the current study are available from the corresponding author upon reasonable request.

## Declarations consent to participate

All listed authors have been approved to participate in the manuscript.

## Declaration of competing interest

The authors declare that they have no known competing financial interests or personal relationships that could have appeared to influence the work reported in this paper.
